# Every Man His Own Dieter

**Published:** 1884-05

**Authors:** 


### EVERY MAN HIS OWN DIETER.

In the biography of the late General Dix is an account of an interview with the celebrated Dr. Abernethy. It will interest our readers, especially since it is known that the wisdom of the physician's advice carried General Dix from dyspeptic youth into eighty years of robust life. General Dix gives the account himself: "He received me with great civility, heard a few words of the story, and cut me short as follows: 'Sir, you are pretty far gone, and the wonder is you are not gone entirely. If you had consulted common sense instead of the medical faculty you could probably have been well years ago. I can say nothing to you ex-

*State a case of sickness in which the law of *similia similibus curantur* was honestly applied, and after failing to cure it, was finally *cured* by some other method of cure.

cepting this: You must take regular exercise, as much as you can bear without fatigue, as little medicine as possible, of the simplest kind, and this only when absolutely necessary, and a moderate quantity of plain food, of the quality which you find by experience best to agree with you. No man, not even a physician, can prescribe diet for another. "A stomach is a stomach;" and it is impossible for any one to reason with safety from his own to that of any other person. There are a few general rules which any man of common sense may learn in a week—such as this: That rich food, high seasoning, etc., are injurious. I can say no more to you, sir; you must go and cure yourself.'"

